# KIR2DS4 Promotes HIV-1 Pathogenesis: New Evidence from Analyses of Immunogenetic Data and Natural Killer Cell Function

**DOI:** 10.1371/journal.pone.0099353

**Published:** 2014-06-05

**Authors:** Aimee M. Merino, Anne-Sophie Dugast, Craig M. Wilson, Paul A. Goepfert, Galit Alter, Richard A. Kaslow, Jianming Tang

**Affiliations:** 1 Department of Medicine, University of Alabama at Birmingham, Birmingham, Alabama, United States of America; 2 Department of Microbiology, University of Alabama at Birmingham, Birmingham, Alabama, United States of America; 3 Ragon Institute of MGH, MIT, and Harvard, Boston, Massachusetts, United States of America; 4 Department of Epidemiology, University of Alabama at Birmingham, Birmingham, Alabama, United States of America; New York University, United States of America

## Abstract

**Background:**

*KIR2DS4* gene variants encode full-length and truncated protein products, with only the former serving as membrane-bound receptors to activate natural killer (NK) cells. We have previously shown that full-length *KIR2DS4* was associated with relatively high viral load and accelerated heterosexual HIV-1 transmission. Our objective here was to provide confirmatory data and to offer new insights about the potential mechanisms.

**Methodology/Principal Findings:**

Mixed models for repeated (longitudinal) outcome measurements on 207 HIV-1 seropositive American youth revealed an association of full-length *KIR2DS4* with relatively high viral load and low CD4^+^ T-cell count (*p*<0.01 for both). Depending on KIR2DS4 expression (presence or absence) on cell surface, NK cells from 43 individuals with untreated, chronic HIV-1 infection often differed in functional properties, including degranulation and secretion of IFN-γ and MIP-1β. In particular, polyfunctional NK cells were enriched in the KIR2DS4-positive subset.

**Conclusions/Significance:**

Full-length KIR2DS4 promotes HIV-1 pathogenesis during chronic infection, probably through the maintenance of an excessively pro-inflammatory state.

## Introduction

As the primary effector cells mediating innate immunity, natural killer (NK) cells are capable of directly destroying virus-infected cells or releasing cytokines to further modulate adaptive immune responses [1]. Several lines of evidence indicate that NK cell functions during HIV-1 infection vary considerably according to the polymorphic nature of killer cell immunoglobulin receptors (KIR) and their natural ligands, especially human leukocyte antigen (HLA) molecules [2], [3], [4], [5]. KIR gene products are stochastically expressed on NK cells and the balance between activating and inhibitory KIRs is critical to cytolysis and other immune function (e.g., cytokine secretion) [6]. In general, inhibitory KIRs are characterized by long cytoplasmic tails (designated by “L” in the gene name) that carry an immunoreceptor tyrosine-based inhibition motif (ITIM), which interacts with Src homology 2–containing tyrosine phosphatases [7]. Activating KIRs have short (S) cytoplasmic tails that mediates interaction with DAP12, a cytoplasmic protein with an immunoreceptor tyrosine-based activation motif (ITAM) [8]. The extracellular portion of KIR molecules consists of 2 (2D) or 3 (3D) Ig-like domains that can bind to HLA-C or HLA-B molecules, respectively [9].

KIR genes have been associated with various outcomes related to organ transplantation, autoimmue disorders and infectious diseases [10], [11]. For example, genotypes encoding KIR3DS1 and KIR3DL1 alleles in combination with their likely ligands (HLA-Bw4) have been associated with protection against HIV-1 disease progression [3], [11], [12]. Among the 15 functional KIR genes clustered at chromosome 19q13.4, *KIR2DS4* defines haplotype A, which has no other genes encoding activating receptors. Truncated forms of KIR2DS4 product lack the transmembrane domain for cell surface expression [13]. In our previous work based on 566 HIV-1 serodiscordant Zambian couples, presence of full-length KIR2DS4 (*001 and *001-like alleles) in chronically infected Zambians was independently associated with accelerated transmission of HIV-1 to cohabiting seronegative partners (RH = 2.00, *p = *0.004), mostly independent of its association with higher (VL) (β = 0.17±0.08, *p = *0.04) [5].

To further illustrate the importance of KIR2DS4 to HIV-1 pathogenesis, we have analyzed 207 HIV-1-infected American youth to provide confirmatory immunogenetic findings. The results were highly consistent with our earlier hypothesis. In addition, we observed that NK cells with and without KIR2DS4 expression often differed in their functional properties, especially in terms of degranulation (production of CD107a) and/or induction of IFN-γ and MIP-1β.

## Results

### Characteristics of HIV-1-infected Youth with Longitudinal Outcome Measures

Among 207 HIV-1-infected youth with 3–4 eligible (treatment-free) visits for this study (**Table 1**), 75.3% were female, and 77.3% self-identified as African American. Their mean age was 18.1 years (range 13.1–21.8), with females being slightly older (18.3±1.2 years) than males (18.0±1.4) (*p* = 0.107). The average VL measured at quarterly follow-up visits was 3.86 log_10_ (range = 0.95–5.49), and the average CD4^+^ T-cell (CD4) count was 475 cells/µL (range = 203–1461). These virologic and immunologic outcomes differed by age and sex (**Table 2**), but not by race (data not shown).

**Table 1:** Characteristics of two study populations with longitudinal and cross-sectional data, respectively.

Baseline characteristics	Subjects with longitudinal data		Subjects with cross-sectional data	
	Full-length *KIR2DS4* (*n* = 160)	Only truncated *KIR2DS4* (*n* = 47)	Full-length *KIR2DS4* (*n* = 23)	Only truncated *KIR2DS4* (*n* = 20)
**Age (years): median and range**	18 (13–22)	18 (14–21)	39 (17–57)	37 (17–50)
**African American: n (%)**	126 (78.8)	34 (72.3)	17 (73.9)	15 (75.0)
**Other races: n (%)**	34 (21.3)	13 (27.7)	5 (21.7)	5 (25.0)
**Female: n (%)**	117 (73.1)	41 (87.2)	11 (47.8)	9 (45.0)
**Male: n (%)**	43 (26.9)	6 (12.8)	12 (52.2)	11 (55.0)
**HIV-1 VL (log_10_): median (range)**	3.8 (1.0–5.5)	3.8 (1.0–5.3)	4.0 (2.0–6.7)	4.0 (2.2–5.6)
**CD4 count: median (range)**	490 (210–1414)	556 (230–1461)	584 (201–990)	577 (203–970)

**Notes**: HIV-1 viral load (VL) in plasma (RNA copies/mL) and CD4^+^ T-cell (CD4) count in peripheral blood (cells/µL) are the two outcomes (see text). The two subgroups with existing PBMC samples share similar features at study entry (*p*>0.45 in all comparisons).

**Table 2:** Associations of full-length *KIR2DS4* gene with longitudinal viral load (VL) and CD4^+^ T-cell (CD4) count in 207 youth with chronic (seroprevalent) HIV-1 infection.

Predictors (independent variables)	Univariable models			Multivariable model	
	VL: Δ ± SE	*P*	*r* ^2^	VL: Δ ± SE	*P*
**Age (per year)**	0.11±0.06	0.013	0.022	0.12±0.08	0.018
**Female sex**	−0.17±0.09	0.049	0.007	−0.09±0.09	0.302
**Full-length ** ***KIR2DS4***	0.29±0.09	0.002	0.011	0.28±0.09	0.002
	**CD4: Δ ± SE**	***P***	***r*** **^2^**	**CD4: Δ ± SE**	***P***
**Age (per year)**	−26±12	0.038	0.015	−24±7	0.015
**Female sex**	66±23	0.003	0.004	50±23	0.001
**Full-length ** ***KIR2DS4***	−64±24	0.008	0.010	−59±24	0.021

**Notes**: The overall *r* ^2^ = 0.039 and 0.033 in the multivariable models for VL and CD4 count, respectively. There is no clear interaction between full-length *KIR2DS4* (gene) and sex (*p* = 0.274).

### Full-length KIR2DS4 as a Correlate of Unfavorable Outcomes in HIV-1-infected Youth

In univariable comparisons of repeated outcome measures, genotypes corresponding to full-length *KIR2DS4* (*001 and *001-like alleles) were associated with relatively high VL (Δ = 0.29±0.09 log_10_, *p = *0.002) (**Table 2**). This association remained true in a multivariable model that adjusted for the effects of age and sex (adjusted Δ = 0.28±0.09 log_10_, *p = *0.002). When conditioned on full-length *KIR2DS4*, the association of female sex with relatively low VL (*p* = 0.010) clearly diminished (adjusted *p* = 0.302 in the multivariable model), but there was no evidence for potential interactions between full-length *KIR2DS4* and sex (*p = *0.274 for the interaction term). The effect of full-length *KIR2DS4* CD4 count was also unfavorable in both univariable (nominal Δ = −64±24, *p = *0.008) and multivariable models (adjusted Δ = −59±24, *p = *0.021) (**Table 2**).

The association of full-length *KIR2DS4* with longitudinal VL and CD4 count did not require the presence of HLA-C*04 that encodes a known ligand for the KIR2DS4 product [14]. For example, VL in 98 youth who had full-length *KIR2DS4* but no HLA-C*04 had higher VL (0.38±0.09 log_10_) than did subjects (*n* = 31) who were doubly negative for full-length *KIR2DS4* and HLA-C*04 (*p*<0.001). Subjects with full-length *KIR2DS4* all had unfavorable outcomes also regardless of the two major HLA-C allele groups (C1 and C2) (*p* = 0.002 to <0.001 in mixed models), being consistent with results reported for HIV-1-infected Zambians [5]. Another reported KIR2DS4 ligand, HLA-A*11 [14], is too rare (<2%) in our study population to allow any meaningful analysis.

### Characteristics of Additional Subjects for Analysis of NK Cell Function

For *ex vivo* and *in vitro* assays, we analyzed existing and cross-sectional samples from 43 adults with chronic HIV-1 infection (**Table 1**). When stratified by *KIR2DS4* genotypes, 23 had full-length alleles, and the remainder (n = 20) had truncated forms only. The two subgroups were highly comparable in terms of: (i) sex ratio (47.8% and 45.0% females, respectively); (ii) ethnic background (*p* = 0.83), and (iii) distribution of other activating KIR genes (*KIR2DS1, KIR2DS2, KIR2DS3, KIR2DS5* and *KIR3DS1* (*p*>0.050) (data not shown).

### KIR2DS4 Expression Profile on NK Cells during Chronic HIV-1 Infection

**Figure 1:**
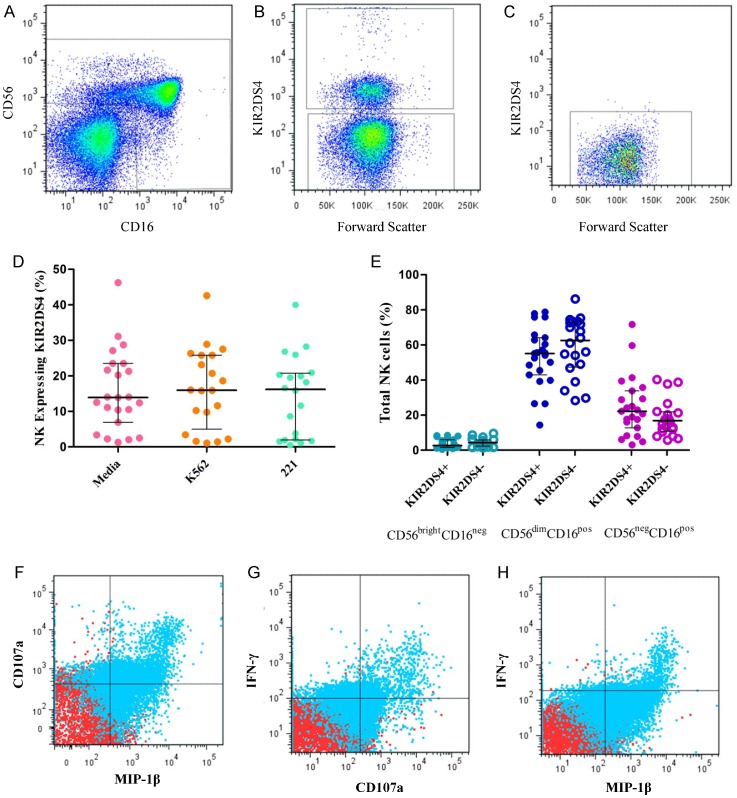
*KIR2DS4* gene expression and natural killer (NK) cell function in subjects with chronic HIV-1 infection. **A**. Flow cytometry gating strategy for NK cells. **B**. Staining of membrane-bound KIR2DS4 on cells derived from subjects with full-length *KIR2DS4* gene (g+), to facilitate the sorting of two populations positive (p+) or negative (p−) for the gene product. **C**. Staining of membrane-bound KIR2DS4 on cells derived from subjects with truncated KIR2DS4 gene only, i.e., negative for full-length *KIR2DS4* gene and gene product (g−/p−). **D**. Percentage of NK cells expressing KIR2DS4 before and after stimulation with HLA-deficient target cells (K562 and 221). **E**. Distribution of NK cell subsets among 43 subjects with and without the full-length *KIR2DS4* genotype. **F–H**. Representative results for staining cell surface CD107a (lysosomal-associated membrane protein 1) and intracellular IFN-γ or MIP-1β before (red) and after (blue) stimulation with K562 cells. In **D** and **E**, the horizontal bars connected by a vertical line correspond to the median and interquartile range.

As expected from stochastic expression of KIRs on NK cells [15], membrane-bound KIR2DS4 receptor was found on a subset of CD3-negative, CD56-positive and/or CD16-positive NK cells in subjects with the full-length *KIR2DS4* gene (i.e., g+); NK cells from subjects without full-length *KIR2DS4* (g−) were naturally all negative for the membrane-bound gene product (p−) (**Figure 1**). For *KIR2DS4* gene-positive (g+) subjects alone, the proportion of NK cells with KIR2DS4 on cell surface varied from 1.28–46.8% (median = 13.9%). NK cells stained positive for KIR2DS4 were predominantly CD3^neg^CD56^dim^CD16^pos^ (median = 85.4%, range = 26.4–100%), with some in the CD3^neg^CD56^neg^CD16^pos^ subset (median = 9.6%, range = 0–59.6%), and very few in the CD3^neg^CD56^bright^CD16^neg^ subset (median = 0.1%, range = 0–1.3%). Stimulation with HLA-deficient cells (K562 or 221) for four hours (effector:target ratio = 10∶1) *in vitro* did not alter the overall proportion of KIR2DS4 expression profile on NK cells (*p* = 0.55 and 0.85, respectively), but activated NK cells did show enhanced degranulation (CD107a staining) and production of IFN-γ and MIP-1β (**Figure 1**). All cell cultures had ≥10-fold increase in NK cell activation (as measured by CD107a staining) after incubation with phorbol 12-myristate 13-acetate (PMA, 2.5 µg/mL) and ionomycin (1 µg/mL).

### KIR2DS4 and NK Cell Function after Stimulation with HLA-deficient Target Cells

**Figure 2:**
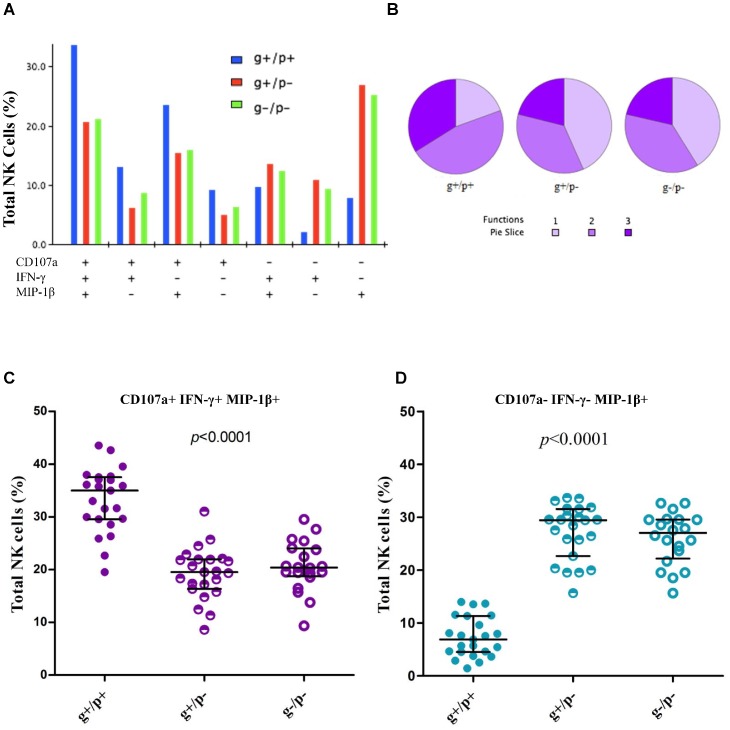
Assessment of polyfunctional profile of natural killer (NK) cells during chronic HIV-1 infection. Results are obtained after stimulation with K562 cells and subtraction of background staining. **A**. Functional properties of NK cells grouped by presence (+) and absence (−) of full-length *KIR2DS4* (g = gene) and membrane-bound KIR2DS4 receptor (p = product), as gauged by production of CD107a, IFN-γ and MIP-1β. **B**. Summary data for mono- and poly-functional NK cells. **C**. Distribution of polyfunctional NK cells. **D**. Distribution of NK cells NK cells producing MIP-1β alone. The g+/p+ and g+/p− cells are derived from individuals carrying full-length *KIR2DS4* gene, while the g−/p− NK cells are derived from individuals with truncated *KIR2DS4* gene only. In **C** and **D**, the horizontal bars connected by a vertical line correspond to the median and interquartile range.

KIR2DS4^+^ and KIR2DS4^−^ NK cells derived from subjects with chronic HIV-1 infection showed differential CD107a, IFN-γ and MIP-1β expression profiles after stimulation with HLA-deficient K562 cells. By all three measurements (individually or in various combinations), NK cells with the full-length *KIR2DS4* genotype (g+) but negative for the KIR2DS4 receptor product (p−) universally behaved like those that were negative for both (g−/p−) (**Figures 2A–2B**). The polyfunctional NK cells co-expressing CD107a, IFN-γ and MIP-1β were highly enriched in the g+/p+ NK cells (median = 35.0%, range = 19.5 to 43.4%) when compared with the g+p− (19.5%) and g−p− NK cells (20.4%) (overall *p*<0.001) (**Figure 2C**). As a single product, MIP-1β was much less frequently expressed (*p*<0.001) by g+/p+ NK cells (median = 6.9%) than by g+/p− (29.4%) and g−/p− cells (27.0% for) (**Figure 2D**).

### Linear Regression of NK Cells Expressing the KIR2DS4 Receptor and Two HIV-1-related Outcomes

**Figure 3:**
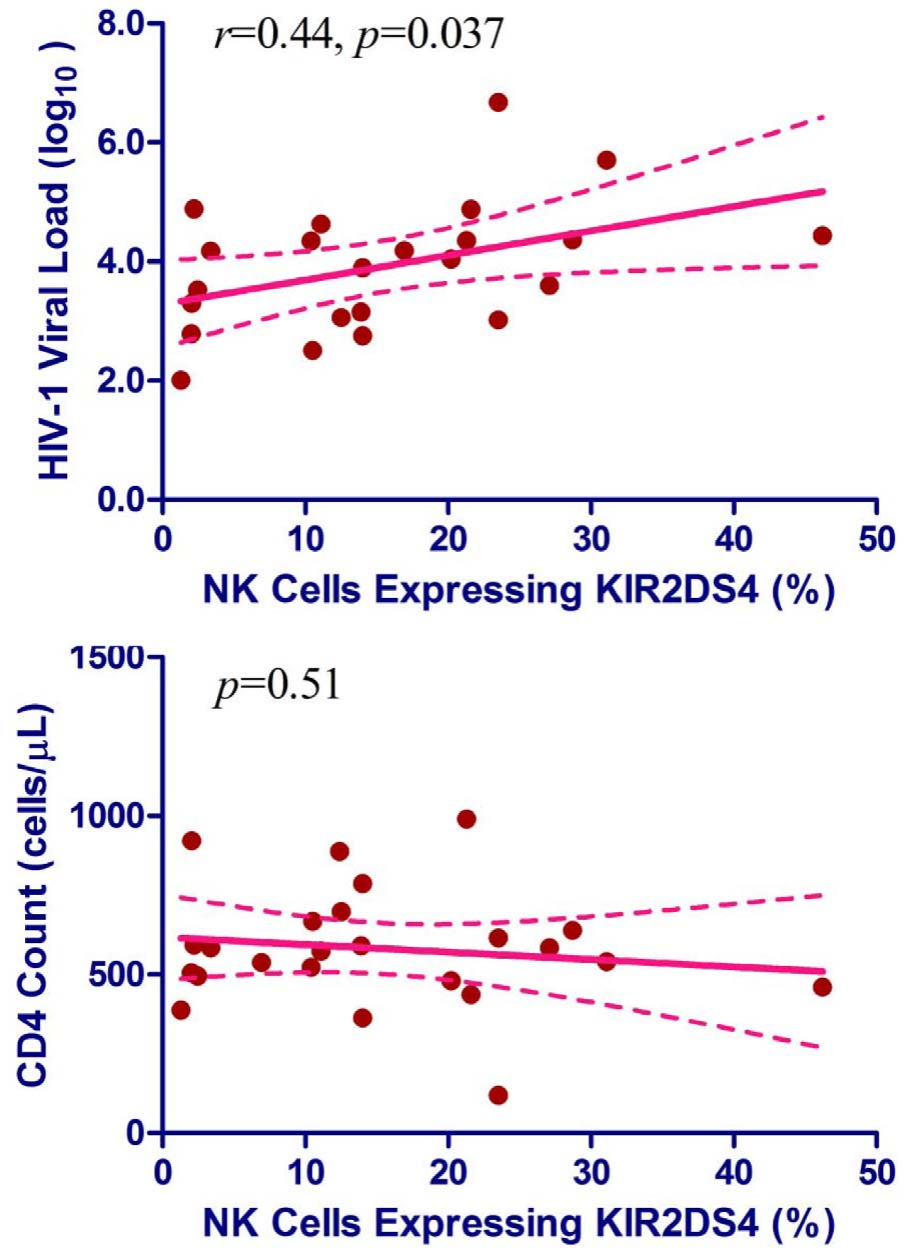
Linear correlation between HIV-1-related outcomes and KIR2DS4 expression in the absence of antiretroviral therapy. Results are based on data from 23 subjects with full-length *KIR2DS4* gene (samples drawn from a single visit). **A**. Correlation of KIR2DS4 expression with plasma HIV-1 viral load (VL) at the time of sampling. **B**. Correlation with CD4^+^ T-cell (CD4) count (cells/µL) at the time of sampling. In each panel, solid and dotted lines correspond to the projected trend line and its 95% confidence intervals, respectively (by linear regression).

In individuals with full-length *KIR2DS4*, the proportion of NK cells expressing the KIR2DS4 receptor showed a linear correlation with log_10_ VL at the time of sampling (*p = *0.037) (**Figure 3A**). The strength of this correlation was modest (Pearson *r* = 0.47, *p = *0.023). However, there was no apparent correlation between the proportion of KIR2DS4^+^ NK cells and CD4 count (*p = *0.51) (**Figure 3B**).

## Discussion

In general, our new findings here are highly consistent with earlier epidemiologic evidence of association between functional, full-length *KIR2DS4* alleles and the likelihood of transmitting HIV-1 infection by chronically infected individuals [5]. The association between full-length *KIR2DS4* and relatively low CD4 count in chronically infected American youth corroborates our original data from HIV-1-infected Zambians who lacked CD4 data. Our North American youth cohort had an additional advantage with longitudinal data before therapy, which improved statistical power when the effects of genetic factors were assessed in mixed models. Beyond demonstrating a consistent relationship between full-length *KIR2DS4* and enhanced HIV-1 pathogenesis in both Africans infected with HIV-1 subtype C viruses and North Americans infected with HIV-1 subtype B, our *ex vivo* and *in vitro* data further point to potential mechanisms for KIR2DS4-related NK cell functions.

KIR2DS4 expression (KIR2DS4 staining) was mostly seen with the CD3^neg^CD56^dim^CD16^pos^ subset of NK cells that are expected to be more cytolytic than the CD3^neg^CD56^bright^CD16^neg^ (KIR2DS4-negative) subset [16]. It is somewhat paradoxical that a KIR gene associated with poor immune control (high VL and low CD4 count) would be associated with a higher proportion of polyfunctional NK cells that are mostly CD3^neg^CD56^dim^CD16^pos^. However, previous reports have shown that HIV-1 infection is associated with enhancement of polyfunctional NK cells [17], while certain combinations of KIR genes and their cognate HLA ligands have been associated with distinct NK cell function [18]. One likely explanation is that prolonged or excessive NK cell activities (as seen in chronic HIV-1 infection) may fuel inflammation and promote HIV-1 pathogenesis. This hypothesis is consistent with the recent notion that elevated levels of pro-inflammatory cytokines and activated NK cells are predictive of HIV-1 acquisition in African women [19]. Alternatively, soluble KIR2DS4 products encoded by truncated alleles could divert or block the function of membrane-bound, full-length KIR2DS4, but this is a less likely alternative hypothesis since the truncated alleles lead to instability of mRNA transcripts [20].

Another likely explanation is the role of IFN-γ, a pro-inflammatory cytokine that has been shown to regulate the transcription of many immune response genes [21], some of which can either contribute to HIV-1 pathogenesis [22] or mediate disease progression [23]. Chronic immune activation during HIV-1 infection can induce IFN-γ responses, which in turn promotes mucosal HIV-1 shedding [24]. These IFN-γ-mediated pathways may partially account for accelerated HIV-1 transmission associated with *KIR2DS4* full-length alleles in Zambians [5]. If KIR2DS4-positive NK cells indeed have improved polyfunctional potential (**Figure 2**), the impact of their function may depend on the timing of the expression or its location within specific tissue compartments [25]. Therefore, until further studies can adequately elucidate the temporal and spatial distribution of NK cell function, the true mechanisms for KIR2DS4-related NK cell function will remain elusive.

*KIR2DS4* has genetic similarities to *KIR3DL2* (encoding an inhibitory receptor) due to gene conversion between the two loci. Thus, in contrast to other activating receptors (e.g., KIR3DS1), KIR2DS4 has gained the ability to bind certain HLA-A motifs while losing some capacity for binding HLA-C ligands [14]. The versatility and/or promiscuity of KIR2DS4 function deserve further investigation, as the ligands for KIR2DS4 have not been fully established. Meanwhile, KIR2DS4 has also been shown to recognize non-HLA ligands [26], probably depending on various environmental factors [27]. The positive correlation between KIR2DS4 expression and HIV-1 VL (**Figure 3**) may well suggest that changes in microenvironments (e.g., severe defects in lymphoid tissues and the gastrointestinal tract) following HIV-1 infection contribute to the observed functional properties of KIR2DS4-positive NK cells.

Within the KIR gene complex, *KIR2DS4* is part of haplotype-A, which carries no other genes encoding activating receptors. It is therefore quite possible that the associations and functional attributes seen with full-length *KIR2DS4* actually reflect the reciprocal roles of neighboring genes that encode various inhibitory receptors. In other words, the relationship of full-length *KIR2DS4* with unfavorable outcomes during chronic HIV-1 infection could be caused by other genes that are inherited as one unit (haplotype-A). This alternative hypothesis would be more likely to be true if full-length and truncated *KIR2DS4* allele tag different subsets of inhibitory KIR gene variants, either in terms of gene contents or expression profiles. Our analyses of KIR2DS4-positive NK cells before and after exposure to HLA-deficient target cells provide some clues. In particular, elevated NK cell activation induced by HLA-deficient target cells, as demonstrated in our experiments, is highly consistent with the anticipated function of inhibitory receptors, i.e., lack of HLA ligands disrupts the function of inhibitory KIR receptors, and more so for the KIR products encoded by haplotype-A (tagged by the *KIR2DS4* gene) than those encoded by other haplotypes with more balanced distribution of activating and inhibitory KIR receptors. In this regard, immunogenetic studies should continue to provide much-needed guidance for follow-up research, especially once fine-mapping for the KIR gene complex reaches a point where it can reliably dissect the effects of alleles and local haplotypes.

In conclusion, full-length *KIR2DS4* and its membrane-bound product appear to have several unique properties related to expression and function within the NK cell pathway. Confirmation of functional KIR2DS4 biomarker of HIV-1 pathogenesis may justify further analysis of direct and indirect roles of KIR2DS4 in NK cell activation and function, especially in the context of the entire haplotype-A of the KIR gene complex. As *KIR2DS4* is outnumbered by several haplotype-A genes encoding inhibitory receptors, the ability of full-length KIR2DS4 to interact with both HLA-A and HLA-C ligands can be an important mechanism for achieving an intrinsic balance between activating and inhibitory receptors. The timing of phenotypic differences between subjects with and without a functional *KIR2DS4* gene also deserves further investigation.

## Subjects and Methods

### Ethics Statement

The research protocols described in this study were approved by the Institutional Review Board at University of Alabama Birmingham (UAB), and all study participants gave written informed consent for participation. Youth participants in the Reaching for Excellence in Adolescent Care and Health (REACH) cohort also received parental permissions when enrolled at various clinical sites [28].

### Study Populations

We first analyzed KIR genotypes in 207 HIV-1 seroprevalent youth (**Table 1**) with retrospective data collected for the REACH project between 1996 and 2000 [28]; subjects with CD4 count <200 cells/µL were excluded in order to minimize confounding by co-infections and other ramifications of end-stage infection [29]. For cross-sectional analysis of NK cell function, we focused on 43 youth and adults with chronic, untreated HIV-1 infection and a relatively recent visit for donating PBMC samples (preserved in liquid nitrogen) needed for *in vitro* assays.

### Plasma HIV-1 Viral Load (VL) and Peripheral Blood CD4 Count as Two Outcomes

Subjects available for association analysis had virologic and immunologic outcomes (VL and CD4 count) for three to four quarterly follow-up visits between 1996 and 2000 [28], [30]. Subjects with existing PBMC samples had cross-sectional data derived from treatment-free visits. For log_10_-transformation, undetectable VLs were assigned a value of 0.5×log_10_ LLD, where LLD is the lower limit of detection in VL assays done by certified laboratories.

### KIR and HLA Genotyping

Using genomic DNA extracted from buffy coat (QIAamp blood kit, Qiagen), we determined KIR gene content and groups of *KIR2DS4* alleles (full-length and truncated) by polymerase chain reaction with sequence-specific primers (PCR-SSP) (Life Technologies/Invitrogen; Grand Island, NY). Methods for molecular HLA class I genotyping have been described elsewhere [5].

### Maintenance of HLA-deficient Cell Lines

Two cell lines devoid of HLA class I expression (K562 from ATCC and 221 from Dr. Peter Parham’s laboratory in Seattle, Washington) were cultured in RPMI-1640 medium supplemented with 2 mM L-glutamine, 100 U/mL penicillin, and 100 U/mL streptomycin.

### Cell Culture and Flow Cytometry Assays

Before experiments, cryopreserved PBMCs were thawed at 37°C and incubated for 6 hrs at a concentration of 1×10^6^ cells/mL of RPMI-1640 medium supplemented with 2 mM L-glutamine, 100 U/mL penicillin, and 100 U/mL streptomycin. Evaluation of NK cell function was done for three sets of PBMCs: (i) in culture medium alone, (ii) stimulation with phorbol 12-myristate 13-acetate (PMA, 2.5 µg/mL) and ionomycin (1 µg/mL) as a positive control, and (iii) co-culture with HLA-deficient K562 or 221 cells at an effector-to-target ratio of 10∶1. The cells were first stained for cell surface markers: (i) CD3 (Pacific Blue), (ii) CD56 (phycoerythrin-Cy7), (iii) CD16 (allophycocyanin-Cy7) (BD Biosciences), (iv) KIR2DS4 (allophycocyanin) (R&D Systems; Boston, MA) and CD107a (phycoerythrin-Cy5) (BD Biosciences; San Jose, CA), all in the presence of GolgiStop (BD Biosciences) and GolgiPlug (BD Biosciences). For further staining of intracellular products, PBMCs were permeabilized with Fix&Perm® (Invitrogen) and then mixed with antibodies specific for IFN-γ (Alexa700) and MIP-1β (phycoerythrin) (BD Biosciences). The stained cells were washed and fixed in 2% paraformaldehyde before sorting using a BD LSRII instrument (BD Biosciences). Compensation was accomplished using BD Comp Beads (BD Biosciences) for each antibody and an unstained cell sample. A minimum of 3×10^5^ events were acquired for each PBMC culture, and the results were analyzed using the FlowJo software (package 7.6.4). Gating was performed with the use of fluorescence-minus-one controls (FMO) and Boolean gating analysis was carried out once positive gates were established for each functional parameter. For quality control, only cells that responded to PMA-ionomycin activation were used for comparative analysis. Of note, results were similar (interchangeable) for PBMCs stimulated with HLA-deficient K562 and 221 cells: therefore, analyses are presented for the experiments with K562 cells. The NK cell gating strategy covered both CD3^−^CD56^+^ and CD3^−^CD56^−^CD16^+^ subsets [31].

### Statistical Analysis of Immunogenetic Data and Flow Cytometry Results

The relationships between full-length *KIR2DS4* and VL or CD4 outcomes were evaluated using mixed models for repeated measures (SAS, version 9.3) (SAS Institute, Cary, NC); non-genetic factors (age, race, and sex) were treated as covariates whenever necessary. The performance of individual models was gauged by their overall *r* ^2^ values (variance explained by factors in the model), while the relative effect of individual factors were determined by the regression beta (mean difference and standard error, SE) and also by the *r* ^2^ values from univariable models. The results from flow cytometry assays were analyzed using GraphPad Prism (version 5) and SPICE (version 5.3033) [32] with a focus on comparing KIR2DS4-positive (+) and KIR2DS4-negative (−) NK cells for positive staining of various immunologic markers and for mean fluorescence intensity (MFI) of immunologic markers. NK cell activity from unstimulated cells was subtracted from the NK cell response after stimulation with target cells to obtain a measure of the net response to HLA-deficient target cells. The Kruskal-Wallis ANOVA test was applied to overall comparisons involving more than two groups, while the Student’s *t*-test was used in comparisons between two groups. The proportion of NK cells expressing KIR2DS4 and HIV-1-related outcomes (VL and CD4 at the time of sampling) was tested using linear correlation (Pearson’s method) and Spearman rank test. In all analyses, statistical significance was accepted at a *p* value of <0.050.
